# Phytate Hydrolysate Differently Modulates the Immune Response of Human Healthy and Cancer Colonocytes to Intestinal Bacteria

**DOI:** 10.3390/nu14204234

**Published:** 2022-10-11

**Authors:** Lidia Hanna Markiewicz, Anna Maria Ogrodowczyk, Wiesław Wiczkowski, Barbara Wróblewska

**Affiliations:** 1 Department of Immunology and Food Microbiology, Institute of Animal Reproduction and Food Research of the Polish Academy of Sciences, Tuwima 10, 10-748 Olsztyn, Poland; 2 Department of Chemistry and Biodynamics of Food, Institute of Animal Reproduction and Food Research of the Polish Academy of Sciences, Tuwima 10, 10-748 Olsztyn, Poland

**Keywords:** phytate hydrolysate, inositol phosphates, colonocytes, gut microbiota, immune response, vegan diet, conventional diet

## Abstract

(1) Phytic acid (PA) is a component of cereal seeds and legumes, therefore its consumption is much higher in a vegan and vegetarian diet compared to a conventional diet. The diet is the main driver of metabolic activity of gut microbiota, therefore, the ability to degrade phytates by the microbiota of vegans significantly exceeds that of the gut microbiota of omnivores. The aim of the study was to investigate the early phase of the immune response of colonocytes treated with an enzymatic hydrolysate of phytic acid (hPA120) and gut bacteria. (2) Cell lines derived from healthy (NCM460D) and cancer (HCT116) colonic tissue and fecal bacteria from vegan (V) and omnivorous (O) donors were investigated. Fecal bacteria were grown in mucin and phytic acid supplemented medium. Cultured bacteria (BM) were loaded onto colonocytes alone (V BM and O BM) or in combination with the phytate hydrolysate (V BM + hPA120 and O BM + hPA120). After a treatment of 2 h, bacterial adhesion, secretion of cytokines, and the expression of genes and proteins important for immune response were determined. (3) All bacteria-treated colonocytes increased the expression of *IL8* compared to controls. The significant increase of the secreted IL-8 (*p* < 0.01) in both cell lines was observed for O BM and O BM + hPA120. The increase of TNF, IL-1β, and IL-10 secretion in healthy colonocytes (V BM alone and with hPA120 treatments; *p* < 0.05) and for TNF and IL-10 in cancer cells (treatments except O BM + hPA120 and V BM, respectively; *p* > 0.05) were stated. A comparison of solely the effect of hPA120 on bacteria-treated colonocytes (BM vs. BM + hPA120) showed that hPA120 decreased expression of *NFkB1* and *TNFR* (*p* < 0.001) in healthy colonocytes. In cancer colonocytes, the expression of *TLR4* and *IL1R* increased after BM + hPA120 treatment, whereas the secretion of IL-8 and *MYD88* and *TNFR* expression decreased (*p* < 0.01). (4) The investigated hPA120 showed a differentiated modulatory activity on the immune response of healthy and cancer human colonocytes. Especially when analyzed independently on the gut bacteria origin, it reduced the proinflammatory response of HCT116 cells to gut bacteria, while being neutral for the bacteria-treated healthy colonocytes.

## 1. Introduction

The intestinal epithelium is a place where effects of endogenous factors (e.g., communication from immune cells, health status, genetic background, host-derived nutrients) overlap with the exogenous (e.g., diet components, degraded food compounds, bacteria and their metabolites), shaping the physiological condition of the epithelium. Diet is a factor impacting both the epithelium and the composition and activity of intestinal microbiota [1]. Microbiota inhabiting the gastrointestinal tract adapts to the environment, therefore a long-term diet can modify its metabolic activity leading to efficient utilization and conversion of diet components [2]. Phytic acid (inositol hexa-phosphate; PA)—the primary storage form of phosphorus in plant seeds—is one of the diet components; the consumption of which is strictly dependent on the dietary pattern, and its consumption in vegan and vegetarian diets is higher compared to the conventional diet [3]. 

Our previous in vitro studies showed that fecal bacteria of vegetarians effectively degraded phytic acid, unlike the fecal bacteria of omnivorous volunteers, which generated mainly tri- and tetra-inositol phosphate (InsP3 and InsP4) derivatives [4]. Inositol phosphates present in the gut lumen can be utilized by bacteria or absorbed by colonocytes. Since inositol phosphates are important second messengers in eukaryotic cells, the composition of PA hydrolysates is a key factor determining their effect on the regulation of cell metabolism and growth [5], as evidenced for 1,2,6-InsP3 [6] and 1,4,5-InsP3 [7].

The site at the colonic lumen and colonic epithelium interface deserves special attention, as this is the place where intestinal components are in the closest proximity to host cells. Bacterial communities located at the intestinal wall and interacting with host cells differ from those inhabiting the lumen of the large intestine [8]. These bacteria have the ability to utilize mucins—glycoproteins secreted by goblet cells that protect colonocytes against direct contact with gut microbiota and induction of inflammatory response. Gut microbiota plays a key role in the development, maturation, and maintenance of a properly functioning intestinal barrier, including the immune status of both the gut and the whole organism [9]. 

Studies describing the effect of single strains on the immune response of intestinal epithelial cells or GALT (gut-associated lymphoid tissue) provide important information about the immunomodulatory potential of these strains, while also pointing to possible mechanisms involved in acquiring tolerance of the organism to commensal bacteria. The effect of a single strain on the development of colon cancer indicated strains of pro- or anti-“cancer” activity [10,11,12]. However, it is studies of the effect of whole microbial consortia on the intestinal epithelium that show the actual net effect of the intestinal microbiota on the immune and physiological status of the gut. The presence of food-derived components in such systems has the potential to modulate the host immune response [13] and, therefore, bring research closer to in vivo conditions. 

In this study, we aimed at investigating whether the early immune response of colonocytes to bacteria is modulated by the hydrolysate of phytic acid. To this end, we used two established cell lines, NCM460D and HCT116, which were derived from heathy and cancer colonic tissue, respectively. The fecal bacteria applied in the study were from vegans and omnivorous volunteers, as diet strongly influences the ability of bacteria to degrade phytate [4]. The fecal bacteria were grown in vitro in the presence of phytic acid and mucin to stimulate the growth of mucin-utilizing and phytate-degrading bacteria. The applied experimental elements were intended to combine three relevant factors acting at the border of colonic epithelium and lumen.

## 2. Materials and Methods

### 2.1. Hydrolysis of Phytic Acid and Determination of the Hydrolysate Composition

Enzymatic hydrolysis of phytic acid (Sigma, Poznań, Poland) was performed as described elsewhere [14] with modifications. The reaction was conducted in a total volume of 5 mL containing 100 mg of phytate and 50 mg of wheat phytase (Sigma, Poznań, Poland) at a pH of 5.15 and a temperature of 55 °C. The reaction was stopped after two hours by heating (100 °C for 5 min). Next, the obtained hydrolysate of phytic acid (hPA120) was cooled down on ice and precipitated enzymatic proteins were centrifuged (12,000× *g*, 10 °C, 10 min). The supernatant was collected, filter sterilized (filter pore 0.22 µm), aliquoted, and stored at −20 °C. Directly before use, the hydrolysate was defrosted and diluted twice with McCoy 5A medium without antibiotics. Before applying onto colonocytes, the hPA120 was 10-fold diluted in a medium appropriate for investigated cell lines.

The composition of inositol phosphates in phytate hydrolysate was determined by applying HPLC-MS (LC-20, Shimadzu, Kyoto, Japan; QTRAP 5500 mass spectrometer, AB SCIEX, Vaughan, Canada) and identified using real standards comprised the retention time and the presence of the respective parent and daughter ion (negative) pairs (Table S1) [15]. 

### 2.2. Volunteers and Cultures of Fecal Bacteria

A total of 3 omnivores (O1–O3) and 3 vegans (V1–V3) (2 women and 1 man in each group, average age O—37.3 ± 6.7, V—36.7 ± 6.6 and BMI—22.0 ± 1.6 and 21.7 ± 2.9, respectively) were donors of fecal samples. Vegan participants followed the vegan diet for three years. Omnivores were on a conventional, western-type diet without any restrictions. None of the participants reported dysfunctions of the digestive tract. The samples were transported to the laboratory at 4 °C within one hour after collection, immediately weighted, portioned, and frozen at −70 °C. All volunteers gave their informed consent for inclusion before they participated in the study. The study was conducted in accordance with the Declaration of Helsinki. Approval No. 2/2016 of the Bioethical Committee at the Faculty of Medical Sciences of the University of Warmia and Mazury in Olsztyn was obtained.

All microbiological analyses were conducted in the anaerobic workstation (MG500; Don Whitley Scientific, Shipley, West Yorkshire, UK; atmosphere composition: 80% N_2_, 10% CO_2_, 10% H_2_). All media, diluents and disposable materials were placed in the workstation at least 24 h before use. Fecal samples (0.1 g) were defrozen in the anaerobic workstation, suspended in 4 mL of peptone water (Oxoid, Basingstoke, UK) and used for inoculation of the modified Wilkins–Chalgren medium. 

Non-selective modified Wilkins–Chalgren medium was prepared as described [4], in short, the medium was supplemented with filter-sterilized phytic acid (Sigma, 1 mM) and with porcine mucin type III (Sigma, 0.5% *w*/*v*). Phytate and mucin were applied in the medium in order to stimulate the growth of bacteria capable of degrading these components, and those that are located at the intestinal wall, close to the mucus layer. Ten milliliters of the medium were inoculated with ca. 10^7^ cells of fecal bacteria from vegan and omnivorous volunteers (samples V1–V3 and O1–O3, respectively) and incubated for 42 h. The exact number of bacterial cells was determined during the adhesion experiment. The non-inoculated medium (CTRL-P) containing all the supplements and proceeded the same way as the inoculated one served as control. 

### 2.3. Characterization of Cultures of Fecal Microbiota

Characterization of bacterial cultures was carried out by separation of polymerase chain reaction products in denaturing gradient gel electrophoresis (PCR-DGGE) according to a protocol by [4] with some modifications. In short, 0.5 mL of the culture was centrifuged (10,000× *g*, 4 °C, 10 min) and the pellet was used for DNA extraction with a Bacterial & Yeats Genomic DNA Purification Kit (Eurx, Gdańsk, Poland). The DNA was used as a template for PCR with 16S rRNA gene-specific primers universal for bacteria (968-GC-f: GC-aacgcgaagaacctta and 1404-r: cggtgtgtacaagaccc) applying conditions specified previously [4]. The obtained amplicons were separated in a polyacrylamide gel with denaturing gradient, stained with SybrGold I (Sigma, Poznań, Poland) and photographed under UV in Gel Doc Imaging System (Bio-Rad, Warsaw, Poland).

### 2.4. Counts of Bacterial Cells

Total counts of bacteria were determined on solid Wilkins–Chalgren medium (Oxoid, Basingstoke, UK) supplemented with 5% of horse blood (Graso, Starogard Gdański, Poland). To this end, samples (cultures of fecal bacteria, control microbiological medium, working solutions of bacteria applied on cell lines, and planktonic and adherent bacteria from the adhesion experiment) were serially diluted in peptone water with NaCl (Oxoid, Basingstoke, UK), plated and incubated for 48 h in the MG500 anaerobic workstation. The bacterial number was expressed as colony forming units (cfu) per milliliter.

### 2.5. Cell Lines and Culturing Conditions

Two cell lines: cancer cells (HCT116; American Type Culture Collection, LGC Standards, Łomianki, Poland) and cells derived from normal colonic tissue (NCM460D; licensed from INCELL Corp., San Antonio, TX, USA) were used as models of colonic epithelium. Cells at passages 21–29 (NCM460D) and 17–35 (HCT116) were used. HCT116 cells were grown in McCoy 5A (Sigma) medium and NCM460D in M3 base medium (INCELL Corp.) at 37 °C, in 96% humidified atmosphere with 5% of CO_2_ (Binder). Media used for routine propagation of the cells were supplemented with FBS (10%; non-heat inactivated fetal bovine serum, FBS, Gibco) and antibiotics (100 µg/mL streptomycin, 100 U/mL penicillin and 25 µg/mL amphotericin B, Sigma). The cells were grown in T75 or T150 flasks to the confluence of approximately 80%, then trypsinized (trypsin-EDTA, Thermo Fisher, Life Technologies) and seeded into new flasks in the ratio of 1:5 (HCT116) or 1:4 (NCM460D). According to the INCELL Corp. recommendations for the proper growth of NCM460D cells, the newly seeded cells were grown in a medium containing approximately 25% of the medium from the previous passage. For the experiment, cells after trypsinization were counted (Scepter, Millipore), seeded onto 6-well (for protein isolation) and 12-well plates (for total RNA isolation and adhesion assay) at a density of 5 × 10^5^ cells/mL (NCM460D) or 3.4 × 10^5^ cells/mL (HCT116) and incubated for 24 h in complete medium with antibiotics. After that time the medium was replaced with a fresh medium without antibiotics and cells were incubated for the next 24 h. Only fully confluent wells were used for the experiment with bacteria. The number of colonocytes in the confluent wells was determined with Scepter Cell Counter (Millipore, Warszawa, Poland) after trypsinization and used for calculation of multiplication of infection (MOI) parameter (a ratio of the bacterial cells number to the number of colonocytes). 

### 2.6. Experimental Design

**Figure 1:**
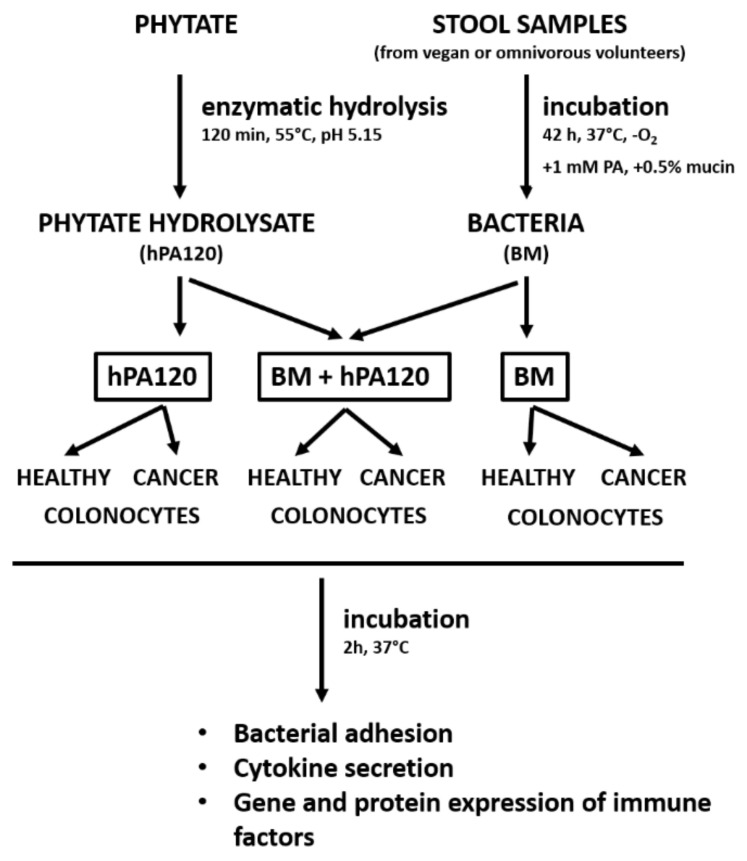
Experimental design.

Two milliliters of cultured fecal bacteria (CFB) (samples O1–O3, V1–V3) and the control medium (CTRL-P) were centrifuged (10,000× *g*, 5 min), then the pellet was washed twice in PBS (incubated in the anaerobic conditions) and suspended in 2 mL of McCoy 5A medium without antibiotics. The bacterial cells were immediately used to prepare working bacterial cells suspensions by adding 100 µL of CFB to 25 mL of McCoy 5A or M3 medium without antibiotics giving finally approximately 8 × 10^6^ bacterial cells per milliliter of the medium. Two types of working bacterial cell suspensions were prepared: BM and BM + hPA120. BM contained only cultured fecal bacteria from vegan (V BM) or omnivorous participants (O BM). BM + hPA120 samples consisted of vegan or omnivorous BM and the phytate hydrolysate hPA120 (V BM + hPA120 and O BM + hPA120, respectively); the concentration of hPA120 was 1 mM as calculated based on the amount of phytate before hydrolysis and its final share in the volume of solutions applied on cells did not exceed 5%. The controls were: colonocytes treated with medium (McCoy 5A or M3 base) without supplements (CTRL), colonocytes treated with medium supplemented with non-inoculated microbiological medium (CTRL-P) processed as the inoculated one; and colonocytes treated with McCoy 5A or M3 base medium containing hPA120 at a concentration of 1 mM. Controls as well as V BM, O BM, V BM + hPA120 and O BM + hPA120 samples prepared in McCoy 5A and M3 were loaded onto HCT116 and NCM460D cells, respectively and incubated for 2 h in conditions appropriate for colonocytes. After that time, supernatants, colonocytes and bacteria were collected for further analyses. A schematic presentation of the experimental design is shown in Figure 1.

### 2.7. Adhesion Assay

After incubation for 2 h, the culture medium was collected and the planktonic bacterial cells along with those released in the further washing steps were plated. Bacterial cells adhering to colonocytes were washed three times with cold PBS and then released by trypsinization (50 µL of trypsin per well, 2 min, 37 °C). Trypsin was inhibited with a medium without antibiotics and the number of adhering bacteria was determined by plating. Based on the obtained counts of planktonic and adherent cells, a percentage of adherent bacterial cells (%ADH) was calculated. 

### 2.8. Cytokine Release in the Post-Culturing Medium

After completion of the experiment, the culture medium was collected, centrifuged (1000× *g*, 1 min), and supernatants were aliquoted, snap frozen in liquid nitrogen, and stored for cytokines and analyses. The profile of inflammatory cytokines (IL-8, IL-1β, IL-6, IL-10, TNF, and IL-12p70) was determined with a BD Cytometric Bead Array (CBA) Human Inflammatory Cytokines Kit (BD Biosciences, Franklin Lakes, NY, USA) and with the use of BD LSR Fortessa Cell Analyzer (BD Biosciences, Franklin Lakes, NY, USA). Obtained results were analyzed with the BD FACS Diva™ version 6.1 software (BD Biosciences, Franklin Lakes, NY, USA). 

### 2.9. Total RNA Isolation, Reverse Transcription and Real-Time PCR

Colonocytes Total RNA was extracted from colonocytes (EXTRACTME Total RNA Kit; Blirt, Gdańsk, Poland), quantified spectrophotometrically and 200 ng was reverse transcribed with TRANSCRIPTME RNA Kit (Blirt). A total reaction volume was 20 µL and included: 2× RT Master Mix containing a combination of random hexamers and oligo(dT)_18_ primers, MgCl_2_ and dNTP mix; TRANSCRIPTME Enzyme Mix containing M-MuLV reverse transcriptase; 200 ng of total RNA and water up to 20 µL. Obtained cDNA was two-fold diluted and used for real-time PCR reactions carried out in Quant Studio 6 Flex (Thermo Fisher Scientific, Warszawa, Poland). Amplifications were performed using gene-specific primer pairs (Table S2) and Fast SYBR™ Green Master Mix (Thermo Fisher Scientific, Warszawa, Poland). Amplification reactions started from heating (95 °C for 20 s) and next 40 cycles of 95 °C for 1 s and 60 °C for 20 s. After each reaction, a melting curve was generated to confirm the specificity of amplicons. Obtained results were analyzed with the Thermo Fisher Scientific Cloud, using a Relative Quantity app, and changes in the expression of tested genes were calculated using the ΔΔC_T_ method using *β-ACT* as a reference gene. 

### 2.10. Western Blot 

Colonocytes were washed twice with cold PBS and lysed in RIPA buffer (Sigma) containing protease inhibitors (Tablets, Sigma) and stored at −70 °C. Protein concentration in cell lysates was determined on Direct Detect assay-free Cards (Millipore, Warszawa, Poland). The two-color detection of proteins was carried out according to [15,16]. In short, proteins were separated on 12% polyacrylamide gels in denaturing conditions, transferred onto a Immun-Blot^®^ Low Fluorescence PVDF membrane (Bio-Rad) and probed with primary antibodies: anti-NF-kB p65 rabbit polyclonal antibody (ab16502; 0.5 µg/mL) and anti-beta actin rabbit polyclonal antibody (ab119716; 1:5000) from Abcam (Cambridge, UK); anti-β-actin (C4) mouse monoclonal antibody (sc-47778; 1:500), anti-TLR2 (A-9) mouse monoclonal antibody (sc-166900; 1:1000), anti-TLR4 (25) mouse monoclonal antibody (sc-293072; 1:1000) and anti-MyD88 (B-1) mouse monoclonal antibody (sc-136970; 1:1000) from Santa Cruz Biotechnology (Dallas, TX, USA). The secondary antibodies were: goat anti-rabbit IgG (H + L) Highly Cross-Adsorbed Secondary Antibody, Alexa Fluor Plus 800 (A32735; 0.05 µg/mL) and goat anti-mouse IgG (H + L) Highly Cross-Adsorbed Secondary Antibody, Alexa Fluor Plus 680 (A32729; 0.05 µg/mL) from Thermo Fisher Scientific. Scans of membranes were obtained in ChemiDoc MP Imaging System (Bio-Rad) and analyzed in Image Lab version 6.0 software (Bio-Rad). 

### 2.11. Statistical Analyses

All statistical analyses were performed with the use of Statistica software v. 13 (StatSoft, Kraków, Polska). A Pearson correlation test was used to evaluate the correlation between MOI and the percentage of adherent cells. The normality of the variables was verified with the Shapiro–Wilk test and homoscedasticity was verified with the Brown–Forsythe test. Due to a lack of normality or homoscedasticity, comparisons of controls and treatments were carried out with the use of a non-parametric Kruskal–Wallis test and applying an embedded module of multiple comparison tests. Evaluation of the effect of hPA120 on bacteria-treated cells (BM vs. BM + hPA120) was performed with a parametric *t* test (variables meeting requirements of normal distribution and variance homogeneity) or with a nonparametric Mann–Whitney U test. 

## 3. Results

### 3.1. Composition of Phytate Hydrolysate

Results of the HPLC-MS analysis of the enzymatic phytate hydrolysate is shown in Table 1. Hydrolysis decreased the amount of IP6 and IP5 by 69% and 25%, respectively, however, they remained the main components of the hPA120 preparation. The next most abundant IPs were IP4 (1,2,3,6-IP4 and 1,2,5,6-IP4), IP2 and IP1 and IP3). The highest increase in concentration was observed for IP1 and IP4, followed by IP3 and IP2 (Table 1). The total amount of the determined inositol phosphates was lower by 41% compared to the non-hydrolyzed preparation, which could be explained by the number of inositol phosphates’ isomers used as real standards in the HPLC-MS analysis, which did not include all possible isomers but focused on those reported to be the main products of enzymatic phytate hydrolysis with wheat phytase [14,17]. There is also the possibility of degradation to previously uncharacterized inositol derivatives. 

### 3.2. Cultures of Fecal Bacteria

The obtained PCR-DGGE banding patterns were characterized by more than 15 bands (Figure S1) visible in each analyzed sample. Considering that the PCR-DGGE method enables semi-quantitative analysis of complex bacterial populations and that only the most abundant taxa can be observed as band(s) in DGGE patterns [18], the obtained profiles indicate a high diversity of cultured fecal bacteria. The comparison of the DGGE profiles did not reveal differences in the dominant bacterial populations characteristic only for group V or O. 

### 3.3. Adhesion of Colonic Bacteria to Cancer and Healthy Colonocytes

In our study, the determined average MOI value was 12.43 ± 8.3 (ranged from 5.75 to 34.7). There was no significant correlation between MOI and the percentage of adhering bacteria (%ADH; *r* = −0.2124, *p* = 0.319), therefore it has been assumed that the observed differences in adhesion were not due to variable proportions of colonocytes and bacterial cells.

**Figure 2:**
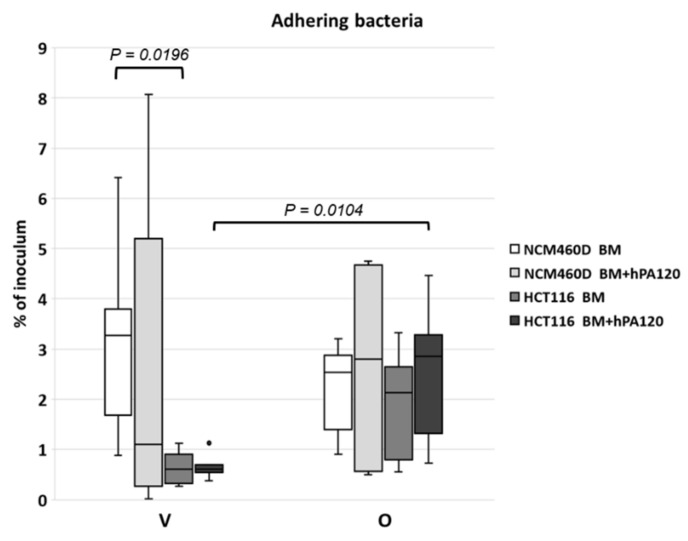
Percentage of adherent bacterial cells (%ADH) to the healthy (NCM460D) and cancer (HCT116) colonocytes. Fecal bacteria derived from vegan (V) and omnivorous (O) volunteers were grown in Wilkins–Chalgren medium supplemented with 0.5% of mucin and 1 mM of phytic acid for 42 h at 37 °C in an anaerobic atmosphere. The adhesion assay was performed with bacteria alone (BM) or with bacteria and the hydrolysate of phytic acid (BM + hPA120), for 2 h at 37 °C. Whiskers—non-outliers, box—25–75% non-outliers; horizontal line in the box—median; black dot—outliers. Significant differences were calculated with the Mann–Whitney U test.

In general, the observed adhesion of fecal bacteria to NCM460D cells ranged from 0.02% to 8.07% (average 2.71% ± 2.27) and was significantly higher (*p* < 0.05) compared to the values recorded for HCT116 cells (0.27% to 4.47%, average 1.41% ± 1.2). A detailed comparison of adhesion showed that %ADH of the same bacteria to different cell lines was significantly higher for vegan BM adhering to NCM460D than to HCT116 (*p* = 0.0196, Figure 2). There were no significant differences in %ADH for vegan BM + hPA120 samples although a tendency for higher %ADH to NCM460D was observed. Bacteria from the omnivorous group adhered with comparable %ADH to both tested cell lines independently on the hPA120 presence (Figure 2). 

### 3.4. Does the Origin of Bacteria and/or the Presence of hPA120 Influence on the Immune Response of Healthy and Cancer Colonocytes?

In the first step, we assessed the effect of single and combined effect of fecal bacteria derived from vegan or omnivorous volunteers and the phytate hydrolysate on the immune response of healthy and cancer colonocytes. 

#### 3.4.1. Healthy Colonocytes 

**Figure 3:**
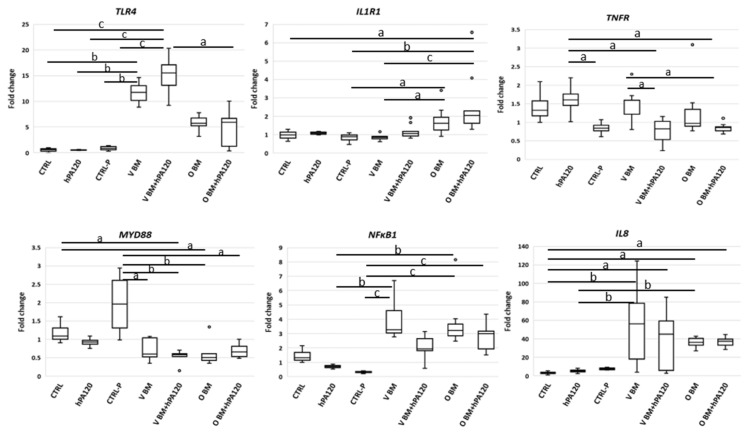
Gene expression in NCM460D cells treated with fecal bacteria derived from vegan (V) or omnivorous (O) volunteers. Fecal bacteria were grown in Wilkins–Chalgren medium supplemented with 0.5% of mucin and 1 mM of phytic acid for 42 h at 37 °C in an anaerobic atmosphere. The treatment was performed with bacteria alone (BM) or with bacteria and the hydrolysate of phytic acid (BM + hPA120), for 2 h at 37 °C. The controls were: non-treated colonocytes (CTRL), colonocytes treated with hPA120 and colonocytes treated with non-inoculated microbiological medium treated as bacterial cultures (CTRL-P). Whiskers—non-outliers, box—25–75% non-outliers; horizontal line in the box—median; circles—outliers. Kruskal–Wallis was applied to calculate significant differences between the treatments, which are marked with: a—values different at *p* < 0.05; b—values different at *p* < 0.01; c—values different at *p* < 0.001.

The analysis showed that NCM460D cells responded to vegan bacteria (samples V BM and V BM + hPA120) with an increase of *TLR4* expression compared to all controls, while the level of this gene in O samples was also elevated but without statistical significance (Figure 3). Only colonocytes treated with omnivorous bacteria (O BM and O BM + hPA1290) increased also the level of *IL1R1* mRNA in relation to CTRL, CTRL-P or V BM. The expression of *NFκB1* increased in all bacteria-treated samples (except for V BM + hPA120) compared to hPA120 and/or CTRL-P controls (Figure 3). It should be noted that CRTL-P (non-inoculated microbiological medium) was the only one that significantly stimulated the expression of the *MYD88* over all the BM-treated samples, however, non-significantly if compared to CTRL and hPA120. On the other hand, all BM and BM + hPA120 samples showed reduced expression of *MYD88*, the statistical significance (*p* < 0.05), however, was confirmed only for V BM-hPA120 and O BM samples. Only the gene expression of *IL8* was significantly increased in all bacteria-treated samples. 

**Figure 4:**
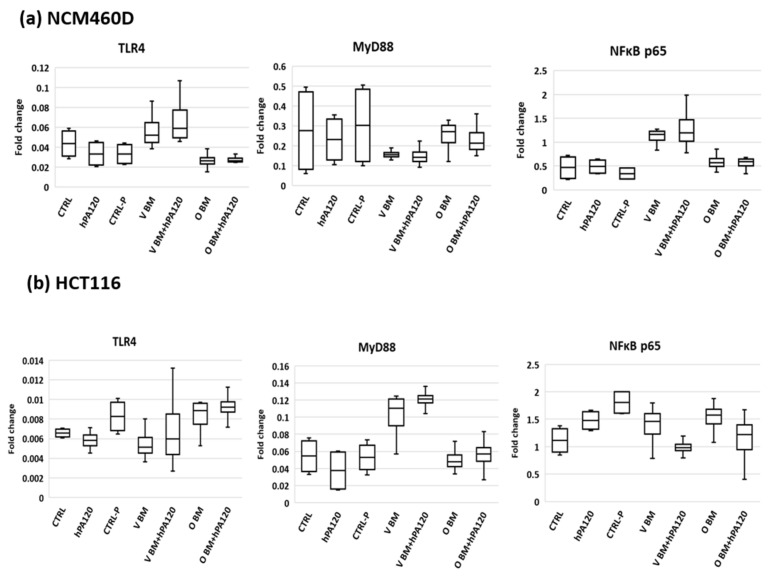
Protein expression of TLR4, MyD88 and NFκB p65 in (**a**)—healthy (NCM460D) and (**b**)—cancer (HCT116) colonocytes. Fecal bacteria derived from vegan (V) and omnivorous (O) volunteers were grown in Wilkins–Chalgren medium supplemented with 0.5% of mucin and 1 mM of phytic acid for 42 h at 37 °C in an anaerobic atmosphere. The treatment was performed with bacteria alone (BM) or with bacteria and the hydrolysate of phytic acid (BM + hPA120), for 2 h at 37 °C. The controls were: non-treated colonocytes (CTRL), colonocytes treated with hPA120 and colonocytes treated with non-inoculated microbiological medium treated as bacterial cultures (CTRL-P). Whiskers—non-outliers, box—25–75% non-outliers; horizontal line in the box—median.

Expression of *TNFR* was affected to a smaller extent—significantly lower levels (*p* < 0.05) were found in CRTL-P and in samples treated with a combination of bacteria (both O and V) and hPA120 compared to hPA120 alone (Figure 3). Despite changes at mRNA level, the protein expression of TLR4, MyD88, and NFκB p65 was not affected by the tested treatments during the 2 hours of this experiment (Figure 4a and Figure S2). Among cytokines tested, secretion of IL-8 elevated the most (ranging from 10 to 70 pg/mL) in all bacteria-treated NCM460D cells (BM and BM + hPA120 samples; Figure 4) confirming the stimulation of mRNA expression.

**Figure 5:**
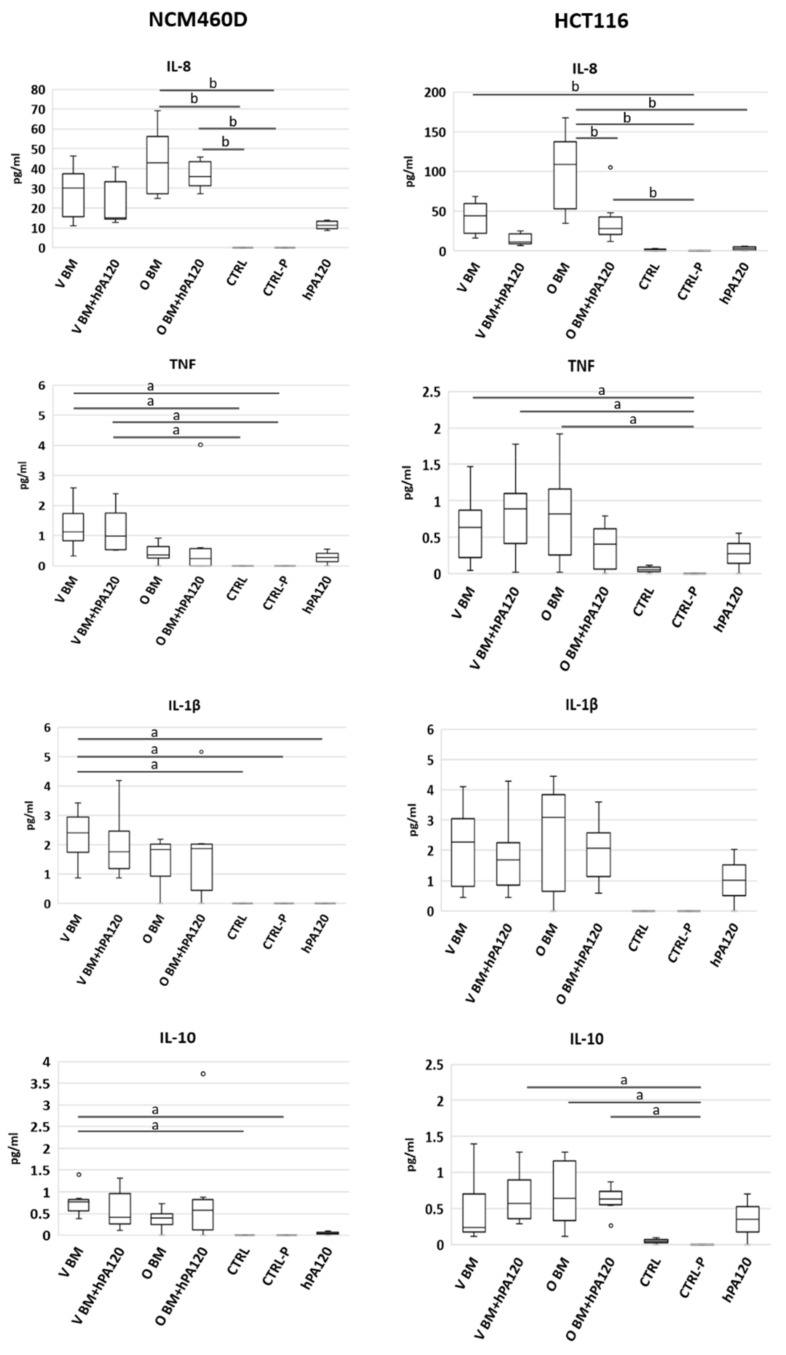
Secretion of interleukin 8 (IL-8), tumor necrosis factor (TNF), interleukin 1β (IL-1 β) and interleukin 10 (IL-10) by healthy (NCM460D) and cancer (HCT116) colonocytes. CTRL—non-treated (control) colonocytes; hPA120—colonocytes treated with phytate hydrolysate; CTRL-P—colonocytes treated with non-inoculated microbiological medium processed as the inoculated one; BM—colonocytes treated with fecal bacteria cultured in Wilkins–Chalgren medium supplemented with 0.5% of mucin and 1 mM of phytic acid for 42 h at 37 °C in an anaerobic atmosphere; BM + hPA120—colonocytes treated with the mixture of BM and hPA120. V or O—fecal bacteria derived from vegan or omnivorous volunteers, respectively. Whiskers—non-outliers, box—25–75% non-outliers; horizontal line in the box—median; circles—outliers. Statistical significance was calculated with the non-parametric Kruskal–Wallis test; a—values different at *p* ≤ 0.05; b—values different at *p* ≤ 0.01, c—values different at *p* ≤ 0.001.

Healthy colonocytes produced the highest amount of IL-8 in response to omnivorous BM applied alone and with hPA120 (*p* ≤ 0.01 compared to controls CTRL and CTRL-P). Secretion of other tested cytokines—TNF, IL-1β, IL-10, IL12p70, and IL-6—was also stimulated in bacteria- and bacteria and hPA120-treated cells, compared to controls; however, their concentration did not exceed 6 pg/mL, while in the CTRL and CTRL-P controls were not detected (Figure 5 and Figure S3). Of notice, only V BM treatment significantly increased the secretion of TNF, IL-1β and IL-10, whereas V BM + hPA120 only increased those of TNF (Figure 5).

#### 3.4.2. Cancer Colonocytes 

**Figure 6:**
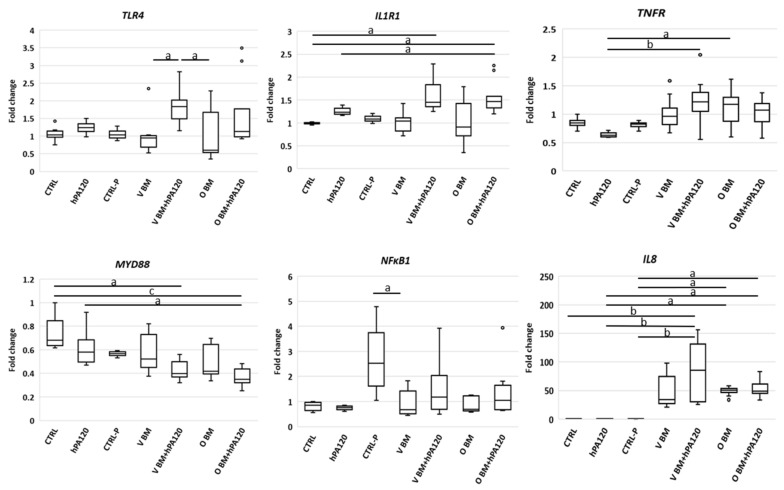
Gene expression in HCT116 cells treated with fecal bacteria derived from vegan (V) or omnivorous (O) volunteers. Fecal bacteria were grown in Wilkins–Chalgren medium supplemented with 0.5% of mucin and 1 mM of phytic acid for 42 h at 37 °C in an anaerobic atmosphere. The treatment was performed with bacteria alone (BM) or with bacteria and the hydrolysate of phytic acid (BM + hPA120), for 2 h at 37 °C. The controls were: non-treated colonocytes (CTRL), colonocytes treated with hPA120 and colonocytes treated with non-inoculated microbiological medium treated as bacterial cultures (CTRL-P). Whiskers—non-outliers, box—25–75% non-outliers; horizontal line in the box—median; circles—outliers. Kruskal–Wallis was applied to calculate significant differences between the treatments, which are marked with: a—values different at *p* < 0.05; b—values different at *p* < 0.01; c—values different at *p* < 0.001.

The cancer colonocytes responded to the presence of the mixture of fecal bacteria and hPA120 (V BM + hPA120 and O BM + hPA120 samples) by an increase of *IL1R1* and a decrease of *MYD88* expression compared to CTRL and/or hPA120 controls (Figure 6). The expression of *TLR4* and *NFκB1* elevated significantly only in V BM + hPA120 (*p* < 0.05 compared to V BM and O BM) and CTRL-P (*p* < 0.05 compared to V BM) samples, respectively. *TNFR* increased significantly in V BM + hPA120 (*p* < 0.01) and O BM (*p* < 0.05) compared only to the hPA120 control. Among analyzed genes, only *IL8* expression significantly increased in all bacteria-treated HCT116 cells (Figure 5), which was accompanied by high secretion of this cytokine (Figure 5). Similarly to NCM460D cells, in HCT116 cancer colonocytes it was the omnivorous BM that generated the highest IL-8 secretion compared to all controls (*p* ≤ 0.01 and *p* ≤ 0.001 compared to CTRL and hPA120, and CTRL-P, respectively; Figure 4). A significant increase in IL-8 secretion by HCT116 cells was also observed in V BM and O BM + hPA120 samples (*p* ≤ 0.01 compared to CTRL-P). Noteworthy, hPA120 tended to decrease the IL-8 secretion when applied in combination with both V and O bacteria. Production of TNF and IL-10 significantly (*p* ≤ 0.05) increased in all treatments except for O BM + hPA120 and V BM, respectively. Unlike the NCM460D cells, HCT116 tended to produce TNF, IL-1β and IL-10 in response to hPA120. On the other side, as in the case of NCM460D cells, in HCT116 colonocytes the protein expression of TLR4, MyD88 and NFκB p65 was not significantly affected by fecal bacteria or their combinations with hPA120 (Figure 4b and Figure S4).

### 3.5. Does the hPA120 Hydrolysate Modulate the Immune Response of Healthy and Cancer Colonocytes to Bacteria?

A comparisons of BM samples vs. BM + hPA120 samples was carried out in order to evaluate whether the partially hydrolyzed phytic acid has a potential to modulate the immune response of healthy and cancer colonocytes to fecal bacteria independently on bacteria’s origin. 

**Figure 7:**
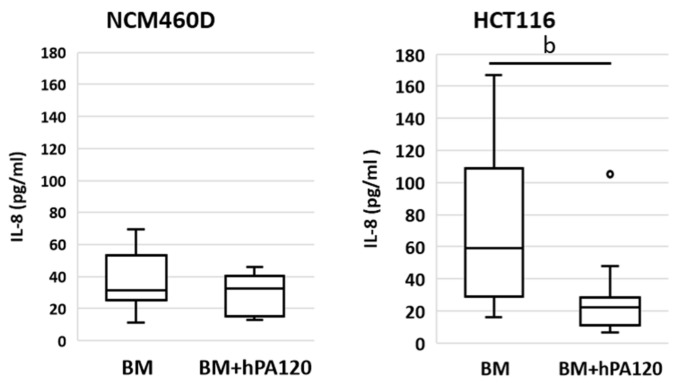
Secretion of interleukin 8 (IL-8) by cancer (HCT116) and healthy (NCM460D) human colonocytes after 2-h incubation with fecal bacterial cultures grown in Wilkins–Chalgren medium supplemented with mucin (BM) and phytate. Colonocytes were incubated with BM alone or BM and phytic acid hydrolysate (BM + hPA120). Whiskers—non-outliers, box—25–75% non-outliers; horizontal line in the box—median; circles—outliers. Values different at *p* < 0.01 (Mann–Whitney U test) are marked with “b”.

**Figure 8:**
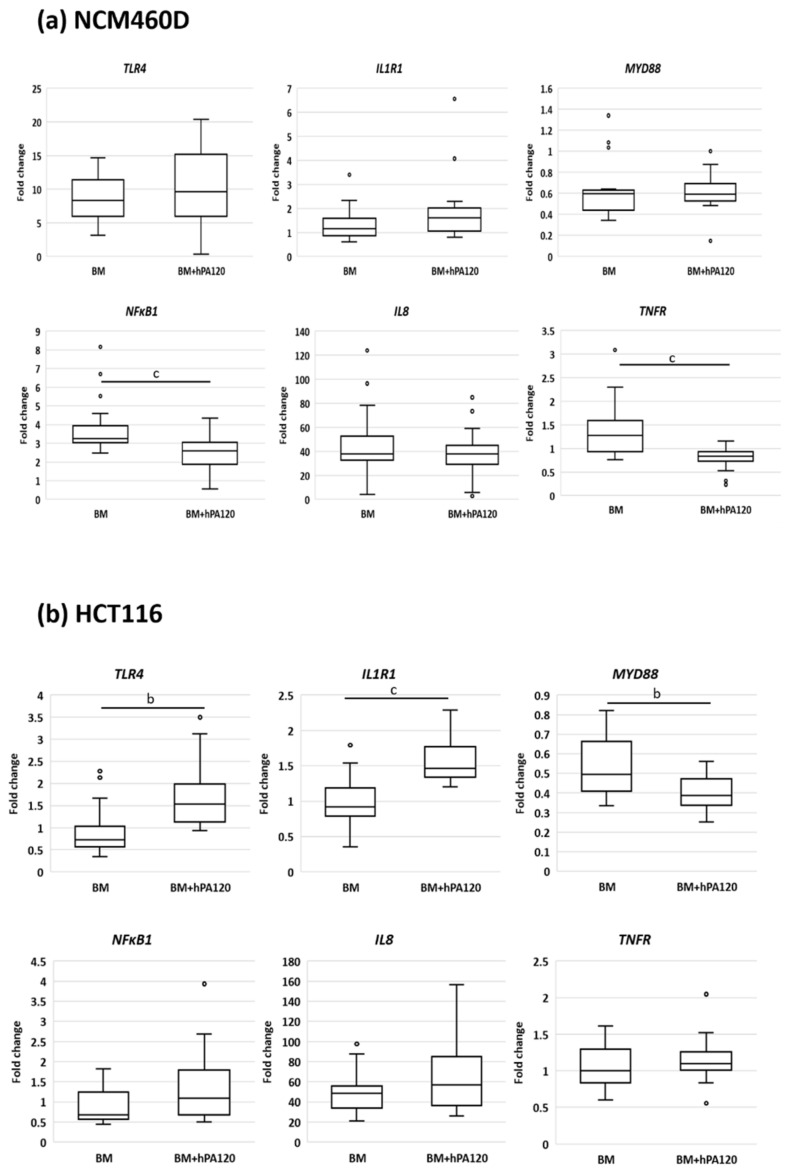
Comparison of the effect of the phytate hydrolysate (hPA120) on gene expression in bacteria-treated (**a**)—healthy (NCM460D) and (**b**)—cancer (HCT116) colonocytes. Fecal bacteria derived from vegan or omnivorous volunteers were grown in Wilkins–Chalgren medium supplemented with 0.5% of mucin and 1 mM of phytic acid for 42 h at 37 °C in an anaerobic atmosphere. Colonocytes were treated with bacteria alone (BM) or with bacteria and the hydrolysate of phytic acid (BM + hPA120), for 2 h at 37 °C. Whiskers—non-outliers, box—25–75% non-outliers; horizontal line in the box—median; circles—outliers. Mann–Whitney U test was applied to calculate significant differences, which are marked with: a—values different at *p* < 0.05, b—values different at *p* < 0.01 or c—values different at *p* < 0.001.

The comparison of the effects of phytate hydrolysate on colonocytes in the presence of colonic bacteria (BM vs. BM + hPA120) revealed differences between normal and cancer colonocytes (Figure 7 and Figure 8). In NCM460D cells, BM + hPA120 reduced the gene expression of inflammation inducing *TNFR* and *NFκB1* compared to cells treated only with bacteria (*p* < 0.001) (Figure 8). In HCT116 cells, hPA1120 stimulated gene expression of TLR4 (*p* < 0.01) and receptor for IL-1β (*p* < 0.001), and lowered the expression of *MyD88* (*p* < 0.01). The comparison of cytokine levels (BM vs. BM + hPA120), revealed that hPA120 significantly (*p* < 0.001) lowered IL-8 secretion only in cancer colonocytes (Figure 7, Figures S4 and S5). There was no significant effect of hPA120 on protein expression of tested factors in both tested cell lines. As we mentioned, the duration of the experiment (2 h) was probably too short for cells to develop a full response at the protein level.

## 4. Discussion

Bacteria are persistently present in the colon in high numbers whereas the content of inositol phosphates (phytic acid and its derivatives) varies depending on a diet (vegan vs. standard diet, type of food products—processed or fresh) and metabolic activity of intestinal microbiota [3,4]. In our study, to obtain a phytate hydrolysate, we used the wheat phytase to bring the experimental conditions close to the real ones in which dietary phytates are partially hydrolyzed by plant phytases of food origin [3]. The investigated hydrolysate contained 31% of IP6 (Table 1) and this amount of intact phytic acid corresponds with the reported degree of phytate degradation in the human gastrointestinal tract ranging from 7 up to 26% [19,20]. What is interesting, the lowest PA degradation degree was observed in young women consuming a low phytate diet, whereas the highest PA degradation was in elderly women consuming a high phytate diet [19,20]. We are aware, that the closest to real conditions would be to apply inositol phosphates generated by colonic bacteria. However, we decided not to use phytate hydrolyzed by colonic bacteria mainly due to almost complete phytate hydrolysis carried out by vegans’ fecal microbiota in vitro and only partial hydrolysis by gut microbiota of omnivorous volunteers [4] that precluded the selection of a reference composition of bacterial phytate hydrolysate. It should be noted that the profile of lower inositol phosphates generated by microbial and plant phytases is different [14], therefore their expected effect on eukaryotic cells can be different.

The process of bacterial adhesion to intestinal epithelium is complex and depends on factors laying at both the bacterial and the host site [21,22]. What is more, in in vitro tests, the final outcome of adhesion assays depends among others on the number of bacteria loaded onto eukaryotic cells [23]. In our study, the determined average MOI value was 12.43 ± 8.3 (ranging from 5.75 to 34.7) and was similar to the routinely applied one [10]. The observed differences in the percentage of adhering bacteria and the extent of intragroup differences (the highest for V BM + hPA120 adhering to NCM460D and the lowest for the same treatment of HCT116 colonocytes; Figure 2) indicated that the presence of phytate hydrolysate had no impact on the mechanisms of bacteria-colonocyte interaction. The observed percentage of adhering bacterial cells, though differentiated among samples and cell lines, was in accordance with values reported for single bacterial strains, which ranged from 0.37–12.2% [24] or 2.45 to 13.5% [25]. 

In this study, we included a control of non-inoculated microbiological medium (CTRL-P), which was to indicate the influence of its components on the response of colonocytes. Some impact of CTRL-P on the healthy and cancer colonocytes was also observed (increased *MyD88* and *NFκB1* expression, respectively) that could indicate the presence in the medium of bacterial or other antigens capable of activating the immune response despite microbiological analysis confirming no viable bacterial cells. It should be stressed, that for a vast majority of the analyzed parameters the response of CTRL-P was comparable to those of the medium control (CTRL).

Maintaining intestinal homeostasis is essential for the host’s health. One of the main components of intestinal homeostasis is a properly functioning intestinal epithelium that ensures the maintenance of the intestinal barrier, the continuity of cell renewal, and protection against bacterial translocation and cancer development. The regulation of these, sometimes contradictory, activities is carried out through immunological mechanisms mainly by the control of pro-inflammatory processes. In the study, we investigated elements involved in the innate immune response at the step of induction (TLR4, IL-1R, TNFR), signaling (MyD88, NFκB) and regulators of immune response (IL-12p70, IL-6, IL-10, TNF, IL-8) [26,27,28,29,30,31,32]. The immunomodulatory activity of phytate was investigated in several in vitro and in vivo models focused mainly on cancer development and progression [33]. Recent studies revealed that phytate alleviated an LPS-induced pro-inflammatory response in mice macrophages [13]. Unfortunately, investigations on the immune effect of phytate hydrolysates or mixtures of inositol phosphates are scarce, therefore this study fills the gap in understanding the function of hydrolyzed phytic acid in the gut environment. 

Phytates are not digested in the human gastrointestinal tract, however, lower inositol phosphates present in the lumen are metabolized or dephosphorylated by gut microbiota. The chemical compounds produced in these processes impact both the microbiota itself and the cells of the intestinal epithelium. Human intestinal epithelial cells (Caco-2 cell line) are capable of dephosphorylation of InsP3 and InsP4, resulting from partial dephosphorylation of phytates by food and microbial phytates [34]. The dephosphorylation ability has not been investigated in NCM460D and HCT116 cell lines yet, therefore we can only speculate that InsP3 and InsP4, as important molecules, are most probably metabolized also by these cell lines. These intermediate products of phytate hydrolysis are also important second intracellular messengers (e.g., Ins(1,4,5)P_3_) that make they are important molecules for a wide spectrum of cellular activities [5]. 

In one of a few studies on phytate derivatives, Wu and Hashimoto-Hill [7] provided in vivo evidence linking commensal bacterial phytase expression, inositol phosphate metabolism and epithelial histone deacetylase 3 (HDAC3) activity in the intestine. HDAC3 represents a convergent epigenetic sensor of distinct metabolites that calibrates host responses to diverse microbial signals. The authors showed the role of gut microbiota and inositol-1,4,5-trisphosphate (InsP3) in the recovery of intestinal epithelium from damage after dextran sodium sulfate treatment [7]. In studies by Ishizuka et al. [35], partially degraded IP3-rich phytic acid suppressed the HCT116 cells proliferation after treatment for 48 h. In our studies, a short (2 h) stimulation with hPA120 alone exerted a minor effect on immune parameters of healthy and cancer colonocytes, as no significant alterations in gene expression, protein level and cytokine release were found, although some increase in the latter case was noted. These changes may indicate that prolonged colonocytes stimulation may reveal the biological activity of the investigated phytate hydrolysate.

Here, healthy colonocytes treated with a mixture of gut bacteria and phytate hydrolysate showed a decrease in the expression of pro-inflammatory factors—*TNFR* and *NFκB1* compared to healthy colonocytes treated only with bacteria (Figure 8). On the other side, cancer colonocytes activated the expression of proinflammatory *TLR4* and *IL1R1* receptors, while reducing *MYD88* in cells treated with the mixture of bacteria and hPA120. These, together with unchanged *NFκB1* expression may indicate either the delayed response of the cancer cells to the applied stimuli or the inhibition of the inflammatory pathway. The latter scenario is supported by significantly lower secretion of IL-8 in HCT116 cells. 

Production of the proinflammatory IL-8 cytokine by intestinal epithelial cells is a physiological response to bacteria and is a part of the resistance mechanism against bacterial invasion that activates the response of immune cells. On the other hand, dysregulated inflammatory response and continual exposure of intestinal tissue to chronic inflammation increase the likelihood of malignant processes [31]. The IL-8 production by intestinal cells in response to bacterial stimuli depends on the specific epitopes present on bacterial surface, such as pathogen-associated molecular patterns (PAMPs) being ligands for TLRs. Activation of different TLRs by complex microbial consortia activates the innate immune response, and the final direction of immune response (pro- or anti-inflammatory) depends on the specific composition of the microbial consortium [36,37,38,39]. In our study, there was no significant difference in IL-8 secretion when compared healthy and cancer colonocytes treated only with V or O bacteria (Figure 3). Moreover, the investigated PA hydrolysate did not disturb the physiological response of healthy colonocytes to intestinal bacteria (unchanged IL-8 secretion), and at the same time showed its anti-inflammatory potential by decreasing the level of the *TNFR* and the regulatory *NFκB* factor (Figure 8). This is extremely important for maintaining both the integrity of the intestinal barrier and preventing chronic inflammation, which can develop as a consequence of prolonged IL-8 production upon bacterial stimulation. On the other hand, in bacteria-treated cancer colonocytes, a strong inhibitory effect of hPA120 on the production of Il-8 was observed (Figure 8). This effect may be crucial for the long-term outcome of the hydrolysate on cancer cells, since the increased production of IL-8 in cancer tissue has been linked with the stimulation of angiogenesis and epithelial-mesenchymal transition (EMT), which, in turn, promotes migration, invasion, and distant metastasis [40]. Nevertheless, the biological effect of the bioactive components of the hydrolyzed phytate remains to be elucidated through comparisons with phytic acid and its individual inositol phosphates derivatives.

The level of IL-8 release in BM-treated HCT116 cells after stimulation for 2 h (av. 60 pg/mL of medium) was at a comparable level as reported for the same cell line after 6 h of stimulation with lactobacilli [37]. The IL-8 mRNA transcription is regulated via the cooperation of inducible (nuclear factor κB) and constitutively expressed (activator protein-1 (AP-1)) transcription factors [31]. Activation of the classical NFκB pathway occurs in response to a variety of host- or pathogen-derived proinflammatory stimuli that bind different receptor families, including toll-like receptors (TLRs), nucleotide oligomerization domain-containing protein 1 and 2, interleukin-1 receptor (IL-1R), and tumor necrosis factor α (TNFα) receptor [31]. Activation of the classical NF-κB pathway was observed in healthy colonocytes, showing increased expression of *TLR4* (strongest in all V BM-treated NCM460D cells), *IL1R1* (strongest in all O BM-treated NCM460D cells), and *NFκB1* and *IL8* (Figure 4). In cancer colonocytes, the activation of pro-inflammatory genes was not so evident compared to the healthy ones, but elevated levels of *TLR4*, *IL1R1* and *TNFα* in some bacteria- and bacteria with hPA120-treated HCT116 cells were found (Figure 5). Surprisingly, no increase of *NFκB1* expression was observed, although, as mentioned before, the increased secretion of IL-8 was present in all bacteria-treated samples. 

The investigated factors did not impact the protein level of the analyzed TLR4, MyD88, and NFκB. The experimental design included incubation of colonocytes with bacterial cells for 2 h, which in many cases is too short a time to develop a full response at the level of both mRNA and protein in eukaryotic cells. In the experimental protocol, a short time of exposition of colonocytes to bacteria was applied due to dynamics in the bacterial counts of bacterial solution during incubation with colonocytes. Fresh cultures of fecal bacteria contained besides strict anaerobes also aerobes such as Proteobacteria (e.g., *E. coli*), which quickly adapt to the aerobic atmosphere and after a lag phase (approximately 2 h) start growing and change the original proportions of aerobes/anaerobes. 

The observed early phase of human colonocytes’ response to bacteria and phytate hydrolysate showed that the hydrolysate has the potential to reduce the secretion of proinflammatory IL-8 in cancer cells while being neutral for the healthy bacteria-treated colonocytes. The attenuation of IL-8 production in cancer cells might be of great importance to human health because this cytokine acts as an autocrine growth factor for colon carcinoma cells and is involved in the development of inflammatory conditions in the gastrointestinal tract [31,41].

## 5. Conclusions

The investigated hydrolysate of phytic acid showed a differentiated modulatory activity on human healthy and cancer colonocytes in the presence of bacteria. However, the observed IL-8 reduction in cancer colonocytes is an optimistic prognosis for further studies on prolonged exposure of colonocytes to the defined phytate hydrolysate and gut microbiota in terms of intestinal inflammation. What is of great importance too, is the proven lack of adverse effects on the immune status of healthy colonocytes providing the rationale for increasing the proportion of plant foods containing PA in the daily diet. 

## Figures and Tables

**Table 1:** Composition of the phytate hydrolysate (hPA120).

Inositol Phosphate *	Content in Non-Degraded Phytate Preparation (μg/mL)	Content in Phytate Hydrolysate (hPA120) (μg/mL)	Change of the Inositol Phosphate Content after Hydrolysis of PA Preparation (%) ^#^
IP6	4352.1	1359.2	31
1,2,3,5,6-InsP5	2524.9	1892.3	75
1,2,3,6-InsP4	29.3	113.9	388
1,2,5,6-InsP4	50.2	498.0	992
1,2,6-InsP3	1.9	14.3	765
1,4,5-InsP3	5.4	6.3	116
1,5,6-InsP3	23.7	31.3	132
1,2-InsP2	11.8	61.4	523
1,4-InsP2	-	-	-
4,5-InsP2	11.8	61.4	523
1-InsP1	1.3	69.3	5447
TOTAL	7012.3	4107.3	59

* Refer to Table S1. ^#^ calculated as % of an inositol phosphate present in non-degraded phytate preparation.

## Data Availability

Data presented in this study are available on request from the corresponding author.

## Supplementary Materials

The following supporting information can be downloaded at: https://www.mdpi.com/article/10.3390/nu14204234/s1, Figure S1: DGGE separation of 16S rRNA amplicons obtained from DNA isolated from bacterial cultures of fecal bacteria obtained from vegan (V1–V3) and omnivorous (O1–O3) volunteers. M—marker of separation distance, Figure S2: Exemplary immunoblots representing detection of indicated proteins in the whole cell extract from NCM460D cells. Figure S3: Secretion of IL-6 and IL-12p70 by healthy and cancer colonocytes (NCM460D and HCT116 cell lines, respectively) after incubation for 2 h with cultures of fecal bacteria alone (BM) or with bacterial cultures and phytate hydrolysate (BM + hPA120). V and O—fecal bacteria obtained from vegan and omnivorous volunteers, respectively. Cultures of fecal bacteria were carried out in modified Wilkins–Chalgren medium supplemented with 1 mM phytic acid and 0.5% mucin, incubated in anaerobic atmosphere, at 37 °C for 40 h. Whiskers—non-outliers, box—25–75% non-outliers; horizontal line in the box—median; empty circles—outliers, Figure S4: Exemplary immunoblots representing detection of indicated proteins in the whole cell extract from HCT116 cells, Figure S5: Concentrations of cytokines released to culture medium by NCM460D cells after 2 h of incubation with BM (human gut bacteria grown in the presence of mucin and phytic acid) or BM and hydrolysate of phytic acid (BM + hPA120), Figure S6: Concentrations of cytokines released to culture medium by HCT116 cells after 2 h incubation with BM (human gut bacteria grown in the presence of mucin and phytic acid) or BM and hydrolysate of phytic acid (BM + hPA120), Table S1: List of the authentic standards used for HPLC-MS analysis of the phytate preparation, Table S2: Primers applied in the study.
