# Supplementary material for: Phytate Hydrolysate Differently Modulates the Immune Response of Human Healthy and Cancer Colonocytes to Intestinal Bacteria
Source: Nutrients. 2022 Oct 11;14(20):4234. doi: 10.3390/nu14204234 (PMC9610475; doi:10.3390/nu14204234)
Supplement: Supplementary file 1 [file nutrients-14-04234-s001.zip › nutrients-1906692-supplementary.pdf]

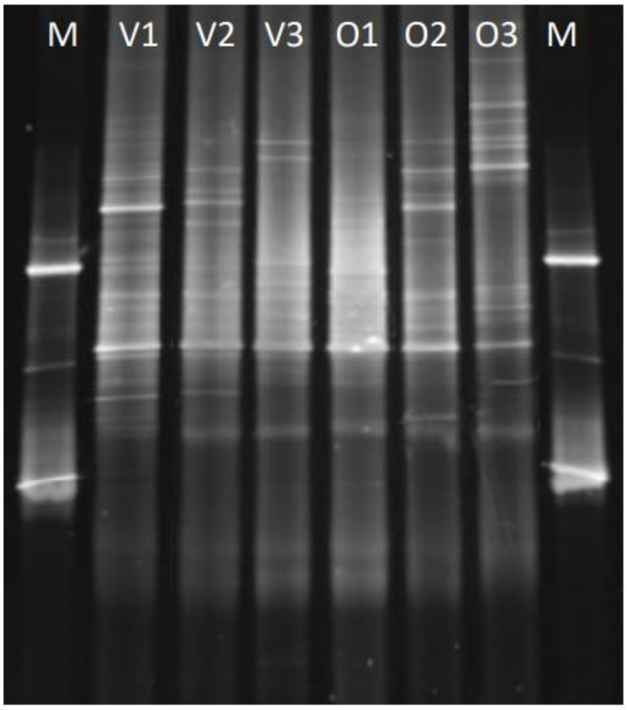

Figure S1. DGGE separation of 16S rRNA amplicons obtained from DNA isolated from bacterial cultures of fecal bacteria obtained from vegan (V1-V3) and omnivorous (O1-O3) volunteers. M – marker of separation distance.

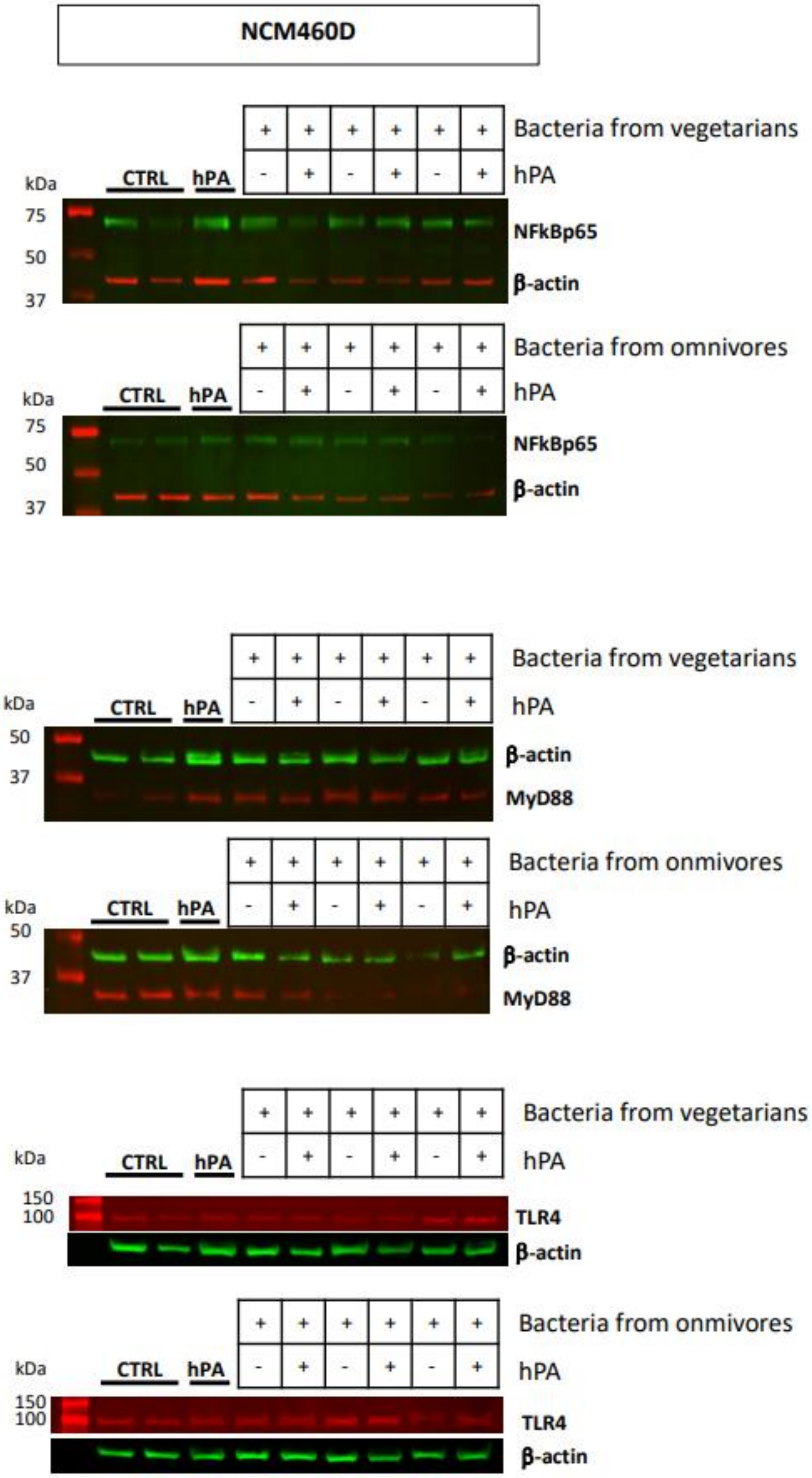

Figure S2. Exemplary immunoblots representing detection of indicated proteins in the whole cell extract from NCM460D cells.

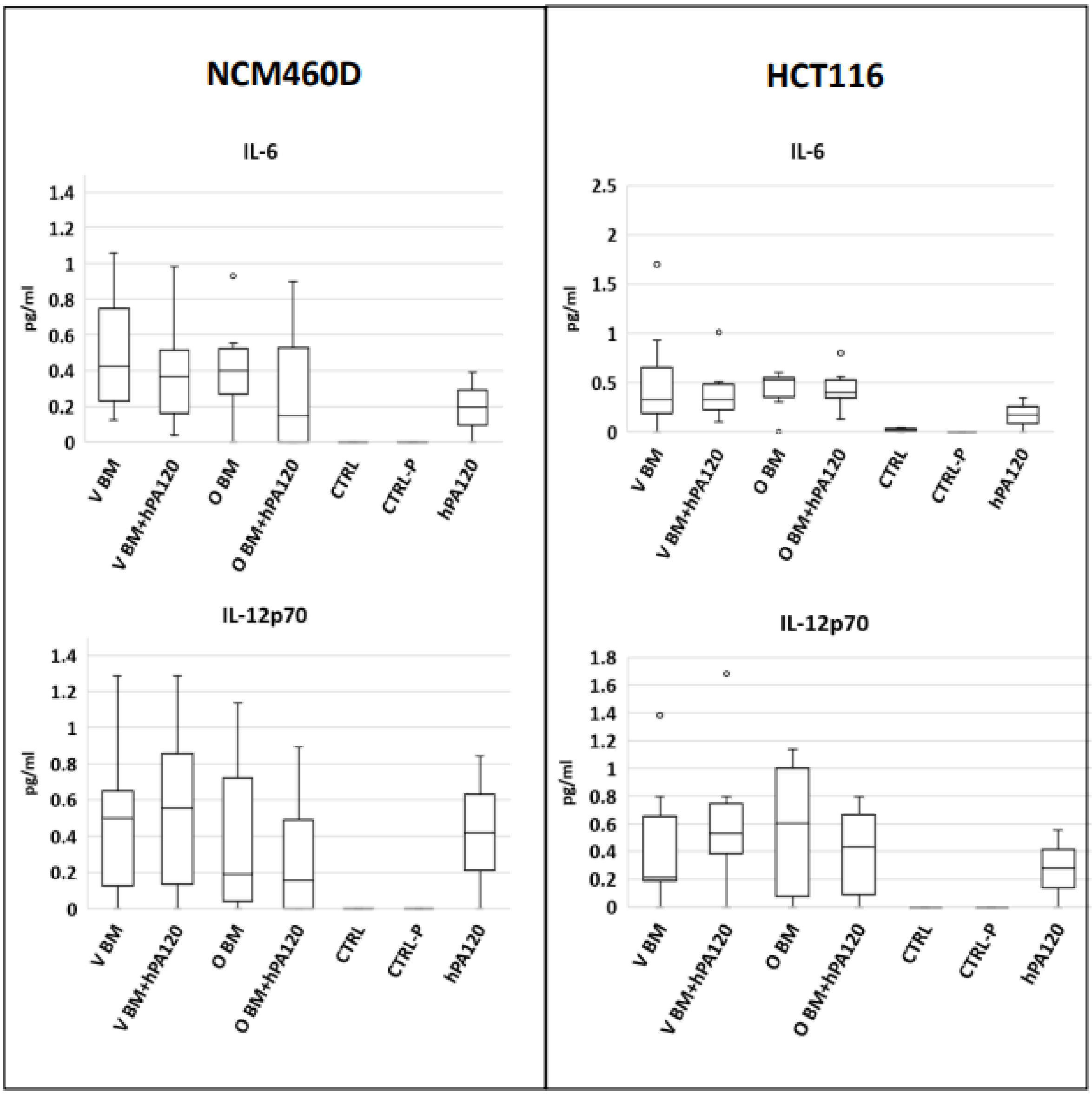

Figure S3. Sectretion of IL-6 and IL-12p70 by healthy and cancer colonocytes (NCM460D and HCT116 cel lines, respectively) after 2 hour incubaction with cultures of fecal bacteria alone (BM) or with bacterial cultures and phytate hydrolysate (BM+hPA120). V and O – fecal bacteria obtained from vegan and omnivorous volunteers, respectively. Cultures of fecal bacteria were carried out in modified Wilkins-Chalgren medium supplemented with 1 mM phytic acid and 0.5% mucin, incubated in anaerobic atmosphere, at 37°C for 40 h. Whiskers – non-outliers, box – 25%-75% non-outliers; horizontal line in the box – median; empty circles – outliers.

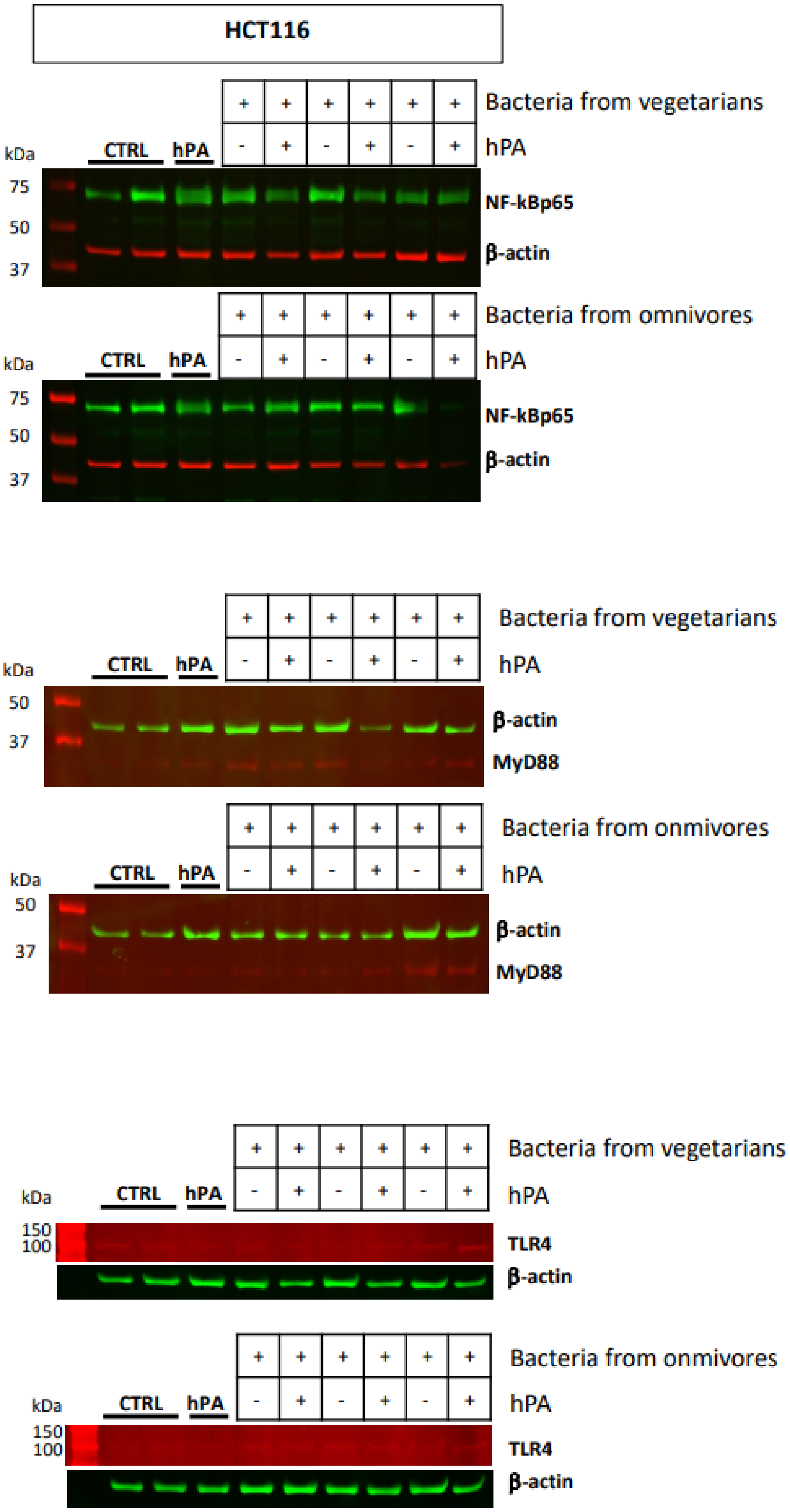

Figure S4. Exemplary immunoblots representing detection of indicated proteins in the whole cell extract from HCT116 cells.

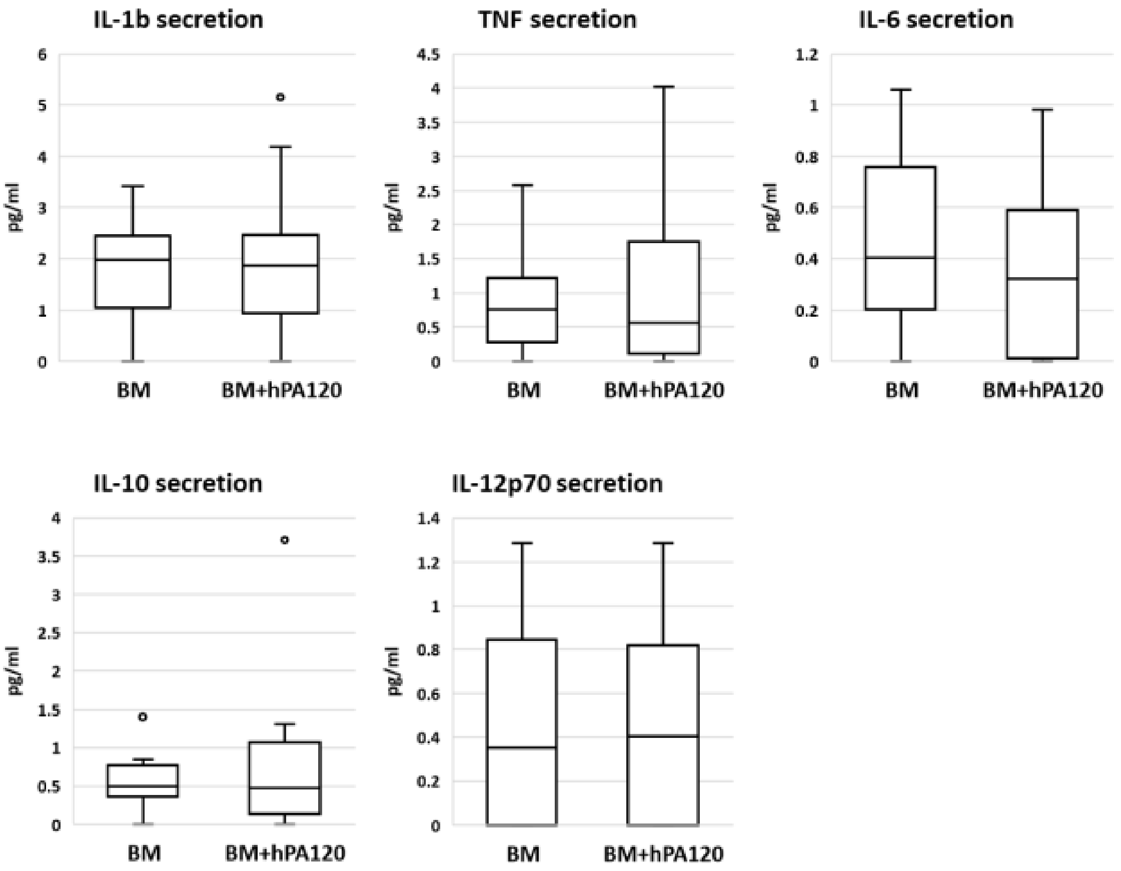

Figure S5. Concentrations of cytokines released to culture medium by NCM460D cells after 2 h incubation with BM (human gut bacteria grown in the presence of mucin and phytic acid) or BM and hydrolysate of phytic acid (BM+hPA120).

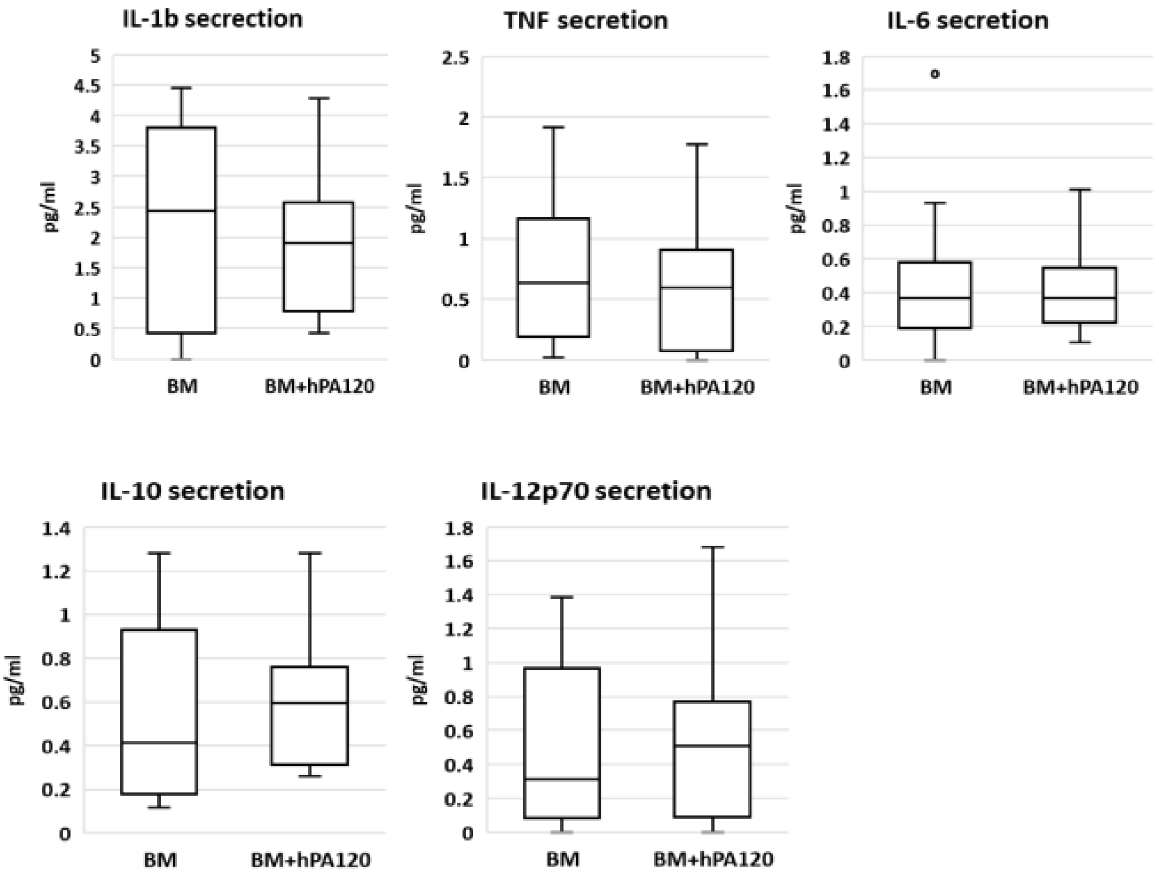

Figure S6. Concentrations of cytokines released to culture medium by HCT116 cells after 2 h incubation with BM (human gut bacteria grown in the presence of mucin and phytic acid) or BM and hydrolysate of phytic acid (BM+hPA120).

Supplementary Information

Table S1. List of the authentic standards used for HPLC-MS analysis of the phytate preparation.

	Name of a standard	Producer, catalog number	Inositol phosphate
1.	D-*myo*-Inositol 1,2,3,5,6-pentakisphosphate (decasodium salt)	Sigma, 76667-1MG	1,2,3,5,6-IP5
2.	D-*myo*-Inositol-1,2,3,6-tetrakisphosphate (tetrapotassium salt)	Cayman Chemical, 10008450	1,2,3,6-IP4
3.	D-*myo*-Inositol-1,2,5,6-tetrakisphosphate (ammonium salt)	Santa Cruz Biotechnology, sc-214815	1,2,5,6-IP4
4.	D-*myo*-Inositol-1,2,6-triphosphate (sodium salt)	Santa Cruz Biotechnology, sc-223918	1,2,6-IP3
5.	D-*myo*-Inositol 1,4,5-trisphosphate (hexapotassium salt)	ENZO Life Sciences, ALX-307-009-M001	1,4,5-IP3
6.	D-*myo*-Inositol-1,5,6-triphosphate (sodium salt)	Santa Cruz Biotechnology, sc-205286	1,5,6-IP3
7.	D-*myo*-Inositol-1,2-diphosphate (sodium salt)	Santa Cruz Biotechnology, sc-223910	1,2-IP2
8.	D-*myo*-Inositol 1,4-bisphosphate (potassium salt)	Santa Cruz Biotechnology, sc-214818	1,4-IP2
9.	D-*myo*-Inositol-4,5-diphosphate (sodium salt)	Santa Cruz Biotechnology, sc-221514	4,5-IP2
10.	D-*myo*-Inositol-1-phosphate (sodium salt)	Santa Cruz Biotechnology, sc-223909	1-IP1
11.	*myo*-Inositol	Santa Cruz Biotechnology, 202714	IP

Table S2. Primers applied in the study.

Name	Sequence (5' → 3')	Target
ACTBf<br>ACTBr	TGAAGTGTGACGTGGACATC<br>ACTCGTCATACTCCTGCTTG	Beta-actin
MyD88-f<br>MyD88-r	GCACATGGGCACATACAGAC<br>TAGCTGTTCCTGGGAGCTGT	Myeloid differentiation primary response 88
NFκB-f<br>NFκB-r	ACAAATGGGCTACACCGAAG<br>TAGGGCTTTGGTTTACACGG	Nuclear factor, light chain / polypeptide, p105
IL-8-f<br>IL-8-r	ACATACTCCAAACCTTTCCACCC<br>CAACCCTCTGCACCCAGTTTTC	Interleukin 8
TNF-R-f<br>TNF-R-r	CACCAGGTGTGATTCAGGTG<br>CCCCACTGTGCTTTGTACCT	Tumor necrosis factor receptor superfamily member 10b (TNFRSF10B)
TLR4-f<br>TLR4-r	TGGACAGTTTCCCACATTGA<br>TGCCATTGAAAGCAACTCTG	Toll-like receptor 4
IL-1βR-f<br>IL-1βR-r	CTG GCA CGT TTG TGA GAA GA<br>GTTCCTTCAAGCAGGCAAAG	IL-1β receptor type I
